# Feimin in Glucose Homeostasis: Mechanistic Insights, Population Heterogeneity, and Therapeutic Potential

**DOI:** 10.2174/0115733998418887250826055154

**Published:** 2025-09-01

**Authors:** Sijun Yang

**Affiliations:** 1 Huangshi Key Laboratory of Birth Defects Prevention, Department of Laboratory Medicine, Huangshi Maternity and Children's Health Hospital, Affiliated Maternity and Children's Health Hospital of Hubei Polytechnic University, Huangshi, 435000, Hubei, People's Republic of China

**Keywords:** Feimin, myokine, glucose homeostasis, diabetes, mer proto-oncogene tyrosine kinase, cytokines

## Abstract

The recent discovery of feimin, a feeding-induced myokine modulating glucose homeostasis *via* the MERTK pathway, offers a novel therapeutic avenue for metabolic disorders. While other studies delineate its acute metabolic effects in Han Chinese populations, critical gaps remain in understanding its mechanistic interplay, population generalizability, and long-term impacts. This perspective synthesizes current evidence, highlights limitations in study design and translational applicability, and proposes integrative approaches, spanning multi-omics, global consortia, and interventional trials, to unravel feimin’s role in metabolic regulation. By addressing these challenges, feimin may emerge as a biomarker or therapeutic target for diabetes and related syndromes.

## 1 INTRODUCTION

The discovery that skeletal muscle secretes cytokines ('myokines') during contraction, has completely changed our understanding of inter-organ communication in metabolic regulation [1]. Among these, the roles of Interleukin-6 (IL-6) and irisin in enhancing glucose uptake and insulin sensitivity have been extensively studied [2, 3]. Recently, Shi *et al.* (2025) identified feimin, a feeding-induced myokine, as a critical modulator of glucose homeostasis *via* the MERTK signaling pathway [4]. This discovery opens new avenues for exploring metabolic disease mechanisms and therapies. However, foundational studies on nutrient-responsive myokines and MERTK's metabolic roles contextualize lingering gaps in feimin's mechanism [5, 6]. The key issues regarding the biological relevance, impact on specific populations, and long-term metabolic effects of feimin remain unresolved. In this study, we synthesize the current cutting-edge evidence in research, emphasize the study's limitations, and propose future directions for advancing Feimin research.

## 2 FEIMIN: A NEW PLAYER IN MYOKINE BIOLOGY

Shi *et al.* demonstrated that feimin binds to the MERTK receptor, with a specific SNP (rs7604639, G>A) in the MERTK gene impairing this interaction and correlating with elevated postprandial glucose levels in Han Chinese individuals. Unlike exercise-induced myokines (*e.g.*, IL-6), feimin is upregulated by feeding, suggesting a unique role in nutrient-responsive metabolic regulation. Mechanistically, feimin-MERTK signaling may intersect with pathways such as Insulin Receptor Substrate (IRS) phosphorylation or AMPK activation, though direct evidence is lacking [4]. Comparative studies with myonectin [7] could clarify whether feimin represents a redundant or complementary regulatory axis. Unlike FGF21, which is primarily liver-derived and targets β-Klotho, feimin’s muscle-specific origin and MERTK dependency may offer tissue-selective therapeutic advantages; however, clinical efficacy of FGF21 analogs is transient, with tachyphylaxis and side effects (*e.g.*, bone loss) at therapeutic doses [8]. Notably, feimin’s muscle-derived, feeding-induced secretion also contrasts with canonical MERTK ligands like Gas6, which drives hepatic insulin resistance by rewiring insulin signaling and increasing insulin receptor trafficking to Endosomes (Table **1**) [9]. While feimin is feeding-induced, its potential modulation by muscle activity, even at low intensities, remains untested. For instance, postprandial autonomic-driven contractions (*e.g.*, gut motility) might synergize with nutrient signals to amplify feimin secretion.

Conversely, exercise-induced myokines, such as irisin, may compete or collaborate with feimin depending on the metabolic context. Furthermore, Shi *et al.* (2025) observed that feimin deficiency elevates plasma triglycerides and hepatic lipid accumulation, implicating its role in dyslipidemia *via* insulin resistance or altered lipid oxidation. Future work should clarify feimin’s direct effects on lipid uptake/lipolysis and assess its therapeutic potential for lipid-driven metabolic disorders. Notably, MERTK is implicated in immune modulation and tissue repair (*e.g.*, fibrosis), yet feimin’s involvement in these processes remains unaddressed. Future work should explore feimin’s systemic effects beyond glucose homeostasis, including immune interactions. A key limitation is the lack of data on non-metabolic consequences, urging caution in therapeutic translation. Resolving these dynamics could unveil feimin as a bidirectional mediator linking nutrient intake and physical activity, with implications for lifestyle interventions in diabetes (Table **1**).

However, translating these promising insights into clinical applications is not without hurdles. In fact, several critical challenges impede the therapeutic translation of feimin research, as highlighted by the foundational studies summarized in Table **2**.

## 3 CHALLENGES IN TRANSLATING FEIMIN RESEARCH

### 3.1 Population Diversity and Genetic Generalizability

By focusing on Southeast Han Chinese, the Shi *et al.* study introduces potential selection bias, as this population's distinct health-conscious behaviors and genetic homogeneity may skew observed metabolic phenotypes.. Notably, the rs7604639 SNP in MERTK is rare in non-East Asian populations (gnomAD), limiting the extrapolation of feimin-MERTK interactions to other ethnic groups [4]. To enhance generalizability, future research must prioritize validation in ancestrally diverse cohorts. Leveraging large-scale biobanks (*e.g.*, UK Biobank) for cross-ethnic meta-analyses of feimin levels and rs7604639 allele frequencies could quantify population-specific effect sizes [10-13]. Collaborative efforts with initiatives like H3Africa and HCHS/SOL would further disentangle gene-environment interactions by examining cohorts with varied dietary habits, physical activity levels, and genetic backgrounds. Such multi-pronged approaches are critical for determining whether feimin-MERTK’s metabolic roles are conserved globally or modulated by ancestry-specific genetic or lifestyle factors [14, 15].

### 3.2 Temporal Dynamics and Causal Inference

The cross-sectional design of Shi *et al.*’s human study precludes causal conclusions. Feimin levels were measured at a single time point, thereby ignoring diurnal variations or chronic exercise effects. Longitudinal cohorts with repeated sampling could elucidate whether feimin fluctuations predict glycemic outcomes. Animal models (*e.g.*, MERTK-knockout mice) are needed to test feimin’s chronic effects on insulin resistance and β-cell function.

### 3.3 Mechanistic Unanswered Questions

The downstream signaling mechanisms of feimin-MERTK, whether it preferentially activates PI3K/Akt or suppresses gluconeogenic genes like PEPCK, remain unresolved, as does its tissue specificity beyond liver and muscle (*e.g.*, adipose or pancreatic actions). To address these dual gaps, mechanistic ambiguity and tissue-specific functions, spatial transcriptomic profiling of metabolic tissues in feimin-overexpressing models would map receptor-ligand interactions (*e.g.*, MERTK expression in adipocytes) and assess pathway activation (*e.g.*, p-Akt or PEPCK levels). Concurrently, CRISPR-based screens in organoids, such as hepatic or adipose tissue models, may clarify whether PI3K/Akt dominance varies across tissues. Furthermore, given parallels to leptin resistance in obesity, the hypothesis that chronic feimin elevation induces MERTK desensitization, such as receptor downregulation or phosphorylation defects, warrants experimental validation. Longitudinal studies in diet-induced obese rodents, combining glucose tolerance tests with phospho-MERTK immunoblotting, would track receptor responsiveness under sustained feimin exposure. Parallel *in vitro* assays using hepatocyte or adipocyte lines treated with escalating feimin doses may quantify temporal signaling attenuation, mirroring paradigms from hormone resistance research. These integrated approaches would systematically unravel feimin’s tissue-specific metabolic interplay and adaptive feedback mechanisms.

## 4 FUTURE DIRECTIONS

Future research on feimin should employ a multi-omic framework to map its interactome and downstream targets comprehensively. Integrating transcriptomic and proteomic profiling with single-cell RNA sequencing of metabolic tissues (*e.g.*, liver, pancreas, visceral adipose) in feimin knockout or transgenic models would reveal spatial expression patterns and receptor-ligand interactions. For instance, combining spatial transcriptomics with MERFISH (Multiplexed Error-Robust Fluorescence *in situ* Hybridization) could resolve whether feimin-producing muscle fibers directly communicate with MERTK-expressing hepatocytes or adipocytes. Spatial transcriptomics may further be paired with viral tracing (*e.g.*, AAV-based retrograde labeling) to delineate feimin’s paracrine (local) *versus* endocrine (systemic) actions across the muscle-liver-adipose axis, thereby clarifying its systemic *versus* localized metabolic roles. CRISPR-based organoid models can resolve tissue-specific MERTK actions, as demonstrated by studies using CRISPR-edited GEJ organoids that revealed proneoplastic features and organoid-based genome screening studies that uncovered novel genes involved in cellular processes [16, 17]. Recent work has demonstrated the utility of CRISPR/Cas9- generated fluorescent reporters in cerebral organoid research, while patient-derived tumor organoid (PDTO) models have enabled kinome-focused CRISPR screens to identify essential kinases [18, 19]. The role of MERTK in organoid systems has been particularly highlighted in studies of efferocytosis regulation and microglial function [20, 21]. Concurrently, interventional studies, such as randomized trials testing feimin supplementation or inhibition in diet-induced obese rodent models—are critical to test therapeutic hypotheses. To ensure global relevance, these efforts must be complemented by consortium-driven collaborations (*e.g.*, harmonizing data across H3Africa, UK Biobank, and HCHS/SOL cohorts) to dissect feimin-MERTK associations across diverse ancestries and lifestyles. Additionally, future studies should elucidate specific nutrient sensors (*e.g.*, GLUT4, INSR) and exosomal regulators (*e.g.*, Rab GTPases) governing feimin secretion, clarifying how feeding signals mechanistically drive myokine release to inform therapeutic targeting. Synergy between multi-omic profiling, spatial biology, functional screening, and translational research would bridge molecular mechanisms with systemic metabolic outcomes, accelerating translational applications.

## CONCLUSION

This perspective synthesizes emerging evidence on feimin, a feeding-induced myokine with therapeutic potential for glucose dysregulation, while identifying critical translational challenges including unresolved mechanistic ambiguities in feimin-MERTK signaling and tissue-specific actions, population heterogeneity that limits generalizability, and uncharacterized temporal dynamics such as postprandial *versus* chronic effects. To address these gaps, we propose targeted strategies encompassing multi-omic spatial profiling to map feimin's interactome, global consortium-driven validation across diverse ancestries, and interventional trials in preclinical models. Collectively, these approaches will elucidate feimin’s role as a precision therapeutic target, accelerating its translation from mechanistic discovery to clinical applications for the management of type 2 diabetes and metabolic syndrome.

## Figures and Tables

**Table 1:** Comparative analysis of feimin and other myokines in metabolic regulation.

**Myokine**	**Induction Factor**	**Secretory Tissue**	**Primary Receptor**	**Key Signaling Pathway**	**Metabolic Function**	**Clinical Associations**	**Controversies/Limitations**	**References**
Feimin	Feeding	Skeletal Muscle	MERTK	PI3K/Akt (hypothesized)	Postprandial glucose homeostasis	MERTK rs7604639 SNP linked to diabetes risk (East Asian populations).	Mechanistic interplay and long-term effects unclear; population generalizability limited.	[4]
IL-6	Exercise	Skeletal Muscle	IL-6R/GP130	JAK/STAT3	Enhances glucose uptake, lipolysis	Marker of chronic inflammation; conflicting roles in insulin resistance.	Pro-inflammatory *vs.* metabolic benefits context-dependent.	[3]
Irisin	Exercise	Skeletal Muscle	αV/β5 integrin	PGC-1α/UCP1	Adipose browning, energy expenditure	Proposed therapeutic target for obesity/T2DM.	Inconsistent circulating levels across studies; unclear clinical translatability.	[2]
Myonectin	Exercise	Skeletal Muscle	LDL receptor family	Undefined	Lipid uptake/storage regulation	Linked to dyslipidemia; mechanisms remain elusive.	Limited evidence for direct metabolic roles.	[7]
FGF21	Fasting/Cold exposure	Liver (primarily)	β-Klotho/FGFR1c	MAPK/ERK	Insulin sensitivity, lipid metabolism	Explored for NAFLD/obesity interventions; species-specific effects.	Transient efficacy in humans; side effects at high doses.	[8]

**Table 2:** Literature review on feimin and comparative myokines.

**Work Carried Out**	**Key Findings/Advantages**	**Limitations/Unresolved Questions**	**References**
Identified feimin as a feeding-induced myokine modulating glucose *via* MERTK; linked rs7604639 SNP to postprandial hyperglycemia in Han Chinese.	First mechanistic characterization of feimin; established clinical-genetic correlation.	Single-ethnic cohort; cross-sectional design; downstream signaling not validated.	[4]
Demonstrated myonectin's role in skeletal muscle protection *via* AMPK/PGC1α.	Elucidated muscle-specific protective pathway; robust *in vivo* models.	Metabolic roles (*e.g.*, lipid regulation) remain unclear.	[7]
Reviewed FGF21's therapeutic potential for metabolic diseases.	Comprehensive clinical translation analysis; multi-tissue targeting.	Transient efficacy; species-specific effects; side effects at high doses.	[8]
Showed GAS6/AXL drive insulin resistance *via* insulin receptor endosomal trafficking.	Revealed novel mechanism of insulin resistance through receptor trafficking.	Limited data on feimin-MERTK interactions or comparative effects.	[9]
Analyzed IL-6's dual roles in inflammation and metabolism.	Context-dependent metabolic benefits (*e.g.*, glucose uptake) well-documented.	Pro-inflammatory effects may overshadow metabolic benefits in chronic disease.	[3]
Studied irisin's effects on adipose browning and energy expenditure.	-	-	[2]

## LIST OF ABBREVIATIONS

AAV: Adeno-Associated Virus
AMPK: AMP-Activated Protein Kinase
CRISPR: Clustered Regularly Interspaced Short Palindromic Repeats
DIO: Diet-Induced Obese
FGF21: Fibroblast Growth Factor 21
GLUT4: Glucose Transporter Type 4
GWAS: Genome-Wide Association Study
IL-6: Interleukin-6
INSR: Insulin Receptor
IRS: Insulin Receptor Substrate
MERTK: Mer Proto-Oncogene Tyrosine Kinase
NAFLD: Non-Alcoholic Fatty Liver Disease
PEPCK: Phosphoenolpyruvate Carboxykinase
PI3K: Phosphoinositide 3-Kinase
SNP: Single Nucleotide Polymorphism
T2DM: Type 2 Diabetes Mellitus
UCP1: Uncoupling Protein 1
